# Unlocking the story of resistance to *Zymoseptoria tritici* in Tunisian old durum wheat germplasm based on population structure analysis

**DOI:** 10.1186/s12864-023-09395-1

**Published:** 2023-06-15

**Authors:** Maroua Ouaja, Bochra A. Bahri, Sahbi Ferjaoui, Maher Medini, Udupa M. Sripada, Sonia Hamza

**Affiliations:** 1 Department of agronomy and plant biotechnology, Laboratory of genetics and cereal breeding (LR14AGR01), The National Agronomic Institute of Tunisia (INAT), University of Carthage, 43 Avenue Charles-Nicolle, Tunis, 1082 Tunisia; 2 Department of Plant Pathology, Institute of Plant Breeding, Genetics and Genomics, University of Georgia, Griffin, GA 30223 USA; 3 Field Crops Laboratory, Regional Field Crops Research Center of Beja (CRRGC), P.O. Box 350, Beja, 9000 Tunisia; 4 Banque Nationale des Gènes (BNG), Boulevard du Leader Yasser Arafat Z. I Charguia 1, Tunis, 1080 Tunisie; 5 International Center for Agricultural Research in the Dry Areas (ICARDA), Avenue Hafiane Cherkaoui, Rabat, Marocco

**Keywords:** Durum wheat landraces, Genetic structure, Admixture, *Zymoseptoria tritici*, Resistance

## Abstract

**Background:**

Septoria tritici blotch (STB) remains a significant obstacle to durum wheat cultivation on a global scale. This disease remains a challenge for farmers, researchers, and breeders, who are collectively dedicated to reduce its damage and improve wheat resistance. Tunisian durum wheat landraces have been recognized as valuable genetic ressources that exhibit resistance to biotic and abiotic stresses and therefore play a crucial role in breeding program aimed at creating new wheat varieties resistant to fungal diseases as STB, as well as adapted to climate change constraints.

**Results:**

A total of 366 local durum wheat accessions were assessed for resistance to two virulent Tunisian isolates of *Zymoseptoria tritici* Tun06 and TM220 under field conditions. Population structure analysis of the durum wheat accessions, performed with 286 polymorphic SNPs (PIC > 0.3) covering the entire genome, identified three genetic subpopulations (GS1, GS2 and GS3) with 22% of admixed genotypes. Interestingly, all of the resistant genotypes were among GS2 or admixed with GS2.

**Conclusions:**

This study revealed the population structure and the genetic distribution of the resistance to *Z. tritici* in the Tunisian durum wheat landraces. Accessions grouping pattern reflected the geographical origins of the landraces. We suggested that GS2 accessions were mostly derived from eastern Mediterranean populations, unlike GS1 and GS3 that originated from the west. Resistant GS2 accessions belonged to landraces Taganrog, Sbei glabre, Richi, Mekki, Badri, Jneh Khotifa and Azizi. Furthermore, we suggested that admixture contributed to transmit STB resistance from GS2 resistant landraces to initially susceptible landraces such as Mahmoudi (GS1), but also resulted in the loss of resistance in the case of GS2 susceptible Azizi and Jneh Khotifa accessions.

**Supplementary Information:**

The online version contains supplementary material available at 10.1186/s12864-023-09395-1.

## Background

Durum wheat (*Triticum. turgidum* L. ssp. *durum*) is a tetraploid species, originated in the Fertile Crescent about 10,000 BP. It evolved from the domestication of hulled tetraploid wheat subspecies as emmer (*Triticum turgidum* L. ssp. *dicoccum*) in the eastern Mediterranean, notably at the mountains of the Fertile Crescent (Iran, Turkey, Syria and Jordan) and at the Tigris and Euphrates basin [1–6]. Thereafter, the geographical expansion of durum wheat had closely followed human migration [4], where Phoenicians, Greeks and Romans contributed crucially in the pervasion and adoption of durum wheat cultivation around the Mediterranean Basin [5]. During their migration, domesticated wheat populations underwent strong natural and human selection processes, after which they have adapted specifically to local environments and developed to become landraces [7, 8]. Howbeit, durum wheat followed two dispersal pathways from its area of origin in the Mediterranean Basin, over the north side via Southern Europe (Turkey, Greece, and Italy) and over the south side via North Africa [1, 9]. The spread of durum wheat populations occurred by land through the Balkans and by maritime route through the Mediterranean Sea [5]. Along these pathways, wheat landraces have traced a complex history of dissemination, adaptation and genetic differentiation in time and space [10]. Therefore, the eastern–western dispersal of the Mediterranean durum landraces favored their divergence into genetically distinct groups. In fact, Moragues et al. [9] classified a collection of 63 durum wheat landraces from the Mediterranean basin in a north dispersal group (including south European and south-western Asian landraces) and a south dispersal group (including landraces from North Africa and the Iberian Peninsula). Soriano et al. [11] also structured a collection of 172 durum wheat landraces from 21 Mediterranean countries into four genetic populations related to their geographical origin, namely eastern Mediterranean, eastern Balkans and Turkey, western Balkans and Egypt, and western Mediterranean.

Durum wheat cultivation history in North Africa and particularly Tunisia, involved the intervention of Phoenicians importing wheat from Lebanon to Carthage, along with the development of Carthage trade maritime activity in the Mediterranean Sea favoring seed exchanges between Tunisia and the Mediterranean countries. North African landraces were also introduced and diffused by Romans, who greatly influenced durum wheat cultivation in this area by setting up modernized irrigation systems [5, 12, 13]. Recently, Robanna et al. [13] studied the structure of six Tunisian durum wheat landraces, and reported their genetic similarity with landraces from North African countries and landraces from Greece, Italy, and Lebanon. Durum wheat was prevalent and well established in North Africa in the classical times [5]. Accordingly, North Africa and Abyssinian regions are considered as secondary centers of diversity for durum wheat [14, 15]. Ren et al. [16] also suggested that North Africa should be considered as a microcenter of wheat diversity. Tunisia, being part of the secondary center of diversity for durum wheat, has a rich collection of old local durum wheat landraces [14]. To date, around 40 old durum wheat landraces are known in Tunisia that were morphologically characterized and classified by Bœuf [14] and Dghais et al. [17]. Several studies emphasised high levels of genetic and agro-morphological diversity [13, 18–22], phenological features [23–26] and resistance to biotic and abiotic stresses i.e. drought, heat and fungal diseases [21, 27–29] within the Tunisian old durum wheat germplasm.

Recently, Ben Krima et al. [30] showed a complex structure of 14 Tunisian durum wheat populations that was not entirely related to their geographic origin and variety name. However, Ouaja et al. [22] identified a strong correlation between the genetic structure of 11 Tunisian durum wheat landraces and their morphological characterisation and nomenclature. Therefore, various interacting factors were reported that have influenced the structure and evolutionary dynamics of durum wheat in Tunisia and the Maghreb region overall, among which complex selection trajectory, the significance of variety names, the occurrence of heterogeneous mixtures within populations, local adaptation, local and regional exchanges between farmers and, loss and misidentification [22, 30, 31]. After the Green revolution, old durum wheat landraces were mainly grown and managed by smallholder farmers under low-input traditional agrosystems in the marginal areas of Mediterranean region, notably in southern Europe and North Africa [4, 11] as they were progressively abandoned from the early 1970s and replaced by improved genetically uniform modern varieties/cultivars [32, 33]. In Tunisia, durum wheat landraces are still cultivated by low-input farmers, in northern and central mountainous areas, under traditional farming systems. These landraces, transmitted by farmers from one generation to the next, are designated by a variety name linked to a historical origin, regional location and specific phenotypic characteristics [22, 30].

A high diversity of Tunisian durum accessions has been observed using morphological descriptors and biochemical markers [22, 34–37]. The genetic diversity of Tunisian durum germplasm was also investigated using different molecular markers such as AFLP and SSR markers, which allowed us to study the genetic variation among and within Tunisian landraces and modern cultivars [20, 22, 38]. Nowdays new high-throughput genotyping technologies such as single nucleotide polymorphism (SNP) arrays or genotyping-by-sequencing (GBS) become a procedure of choice. In fact, based on genotyping, several studies were conducted to analyse genetic diversity and the genetic structure of durum wheat landraces and modern cultivars in the mediteraneen basin and Ethiopia [13, 39–41].

Septoria Tritici Blotch (STB) caused by the fungus *Zymoseptoria tritici* (*Z. tritici*) (Desm.) (formerly *Mycosphaerella graminicola*) is currently considered among the most damaging and worldwide distributed fungal disease on cultivated wheat [42, 43]. The appearance of *Z. tritici* as a host-specialized wheat pathogen occurred about 10,500 years ago via host tracking throughout the time of wheat domestication [44–46]. Comparative genomic analysis between *Z. tritici* and its close relatives highlighted strong adaptive evolution of *Z. tritici* in relation to specialization on wheat [47]. Howbeit, Stukenbrock et al. [44] demonstrated that wheat-adapted *Z. tritici* was derived from an ancestral population infecting wild grasses in the Middle East and that the domestication of an agricultural crop was concomitantly accompanied by the domestication of a fungal pathogen. Accordingly, the Fertile Crescent is considered a hotspot of *Z. tritici* genetic diversity [44, 48]. Therefore, wheat landraces and their wild relatives from the Fertile Crescent, having co-evolved for a long time with *Z. tritici*, must harbor the greatest diversity for resistance to STB [49]. Moreover, a host species specialization was highlighted in *Z. tritici* populations to either bread or durum wheat [50–52] making bread wheat-derived *Z. tritici* strains not suitable to decipher *Stb* genes in durum wheat, as studies into the genetic basis of STB resistance were entirely based on the *Z. tritici*-bread wheat pathosystem [53–58].

In Tunisia and under suitable environmental conditions for infection, STB causes considerable yield losses up to 50–60% [59, 60]. The introduction of the modern cultivar ‘Karim’ in 1980 displaced the cultivation of landraces, resulting in a reduction of the genetic diversity (genetic erosion) and therefore, enhancing the susceptibility to STB [61, 62]. Durum wheat landraces, characterized by a substantial level of genetic diversity [11, 20, 22], represent the main sources of resistance to *Z. tritici* to be incorporated into breeding programs for a sustainable STB disease control and management [21, 63, 64]. The present study consisted on (a) analyzing the genetic structure of 366 Tunisian durum wheat accessions belonging to 13 landraces using 286 single nucloetoide polymorphic markers (SNPs), (b) evaluating the resistance of these accessions to two durum-wheat derived *Z. tritici* isolates (Tun06 and TM220) and, (c) relating genotyping and disease screening data of the Tunisian durum wheat accessions to describe the life history of STB resistance in local durum wheat landraces.

## Results

### Genetic structure of durum wheat landraces

**Fig. 1:**
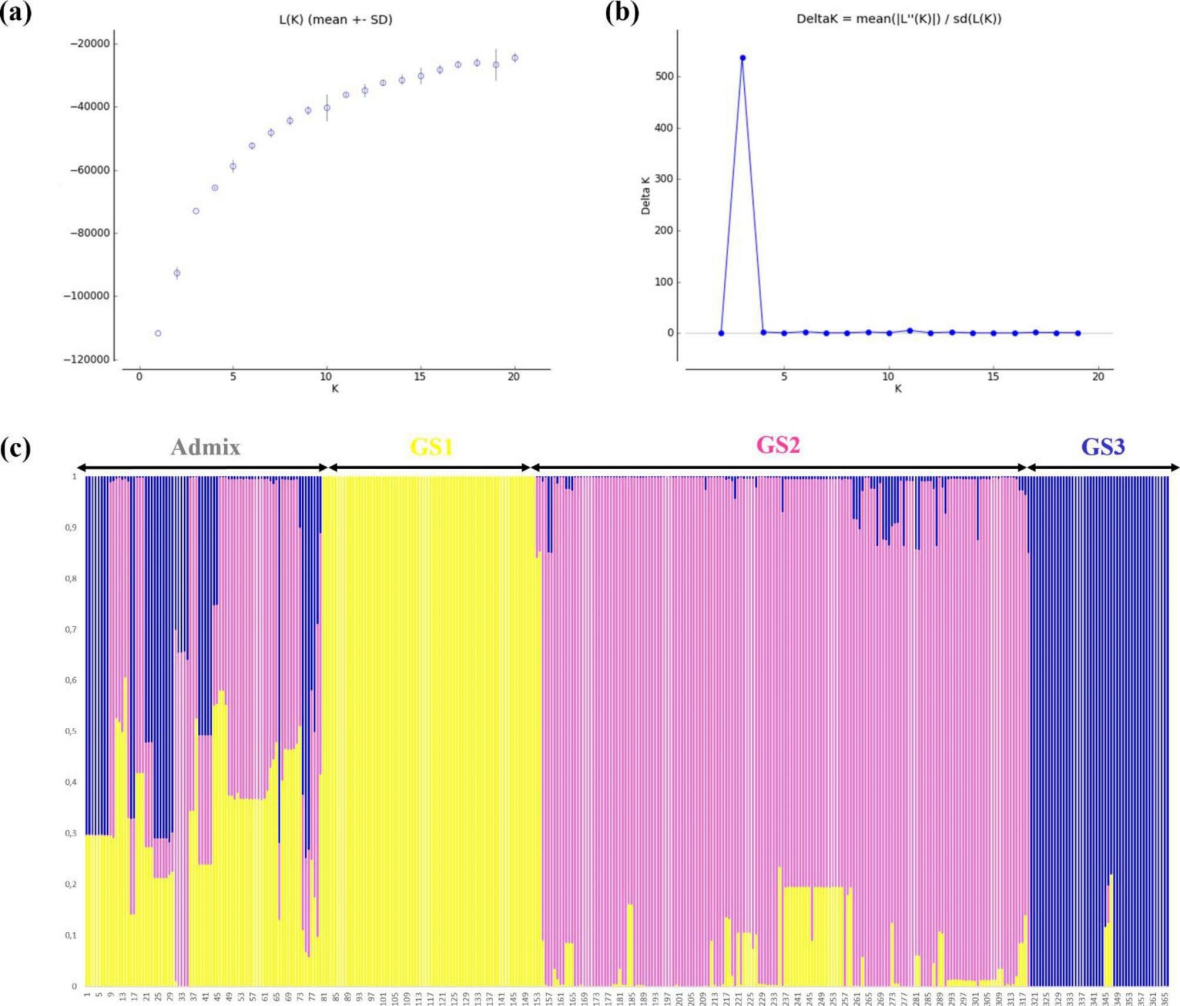
Genetic structure analysis of 366 Tunisian durum wheat accessions genotyped with 286 SNP markers: **(a)** Plot of mean posterior probability (ln P(D)) values per cluster (K); **(b)** delta-K analysis of Ln P(D), for K ranging from 1 to 20; **(c)** Membership coefficient bar plot displaying genetic structure at K = 3 from STRUCTURE [65]. Each genotype is represented by a vertical line

The genetic structure of the durum wheat accessions was determined using the Bayesian clustering model implemented in STRUCTURE [65]. The maximum likelihood (LnP (K)) and delta K (ΔK) methods [66]) showed that the most likely number of genetic subpopulations (K) was 3 (Fig. 1, a and b). The inferred population structure at K = 3 showed that 78% of the genotypes have a membership coefficient higher than 0.7 to one of the genetic subpopulations (GS1, GS2 and GS3), the rest were admixed. GS1, GS2, GS3 and admixed genotypes represented 20%, 45%, 13% and 22% of the entire collection, respectively. Among admixed genotypes, 53%, 27% and 20% were admixed between G1-G2, G1-G3 and G2-G3, respectively (Fig. 1c, Table S1).

**Fig. 2:**
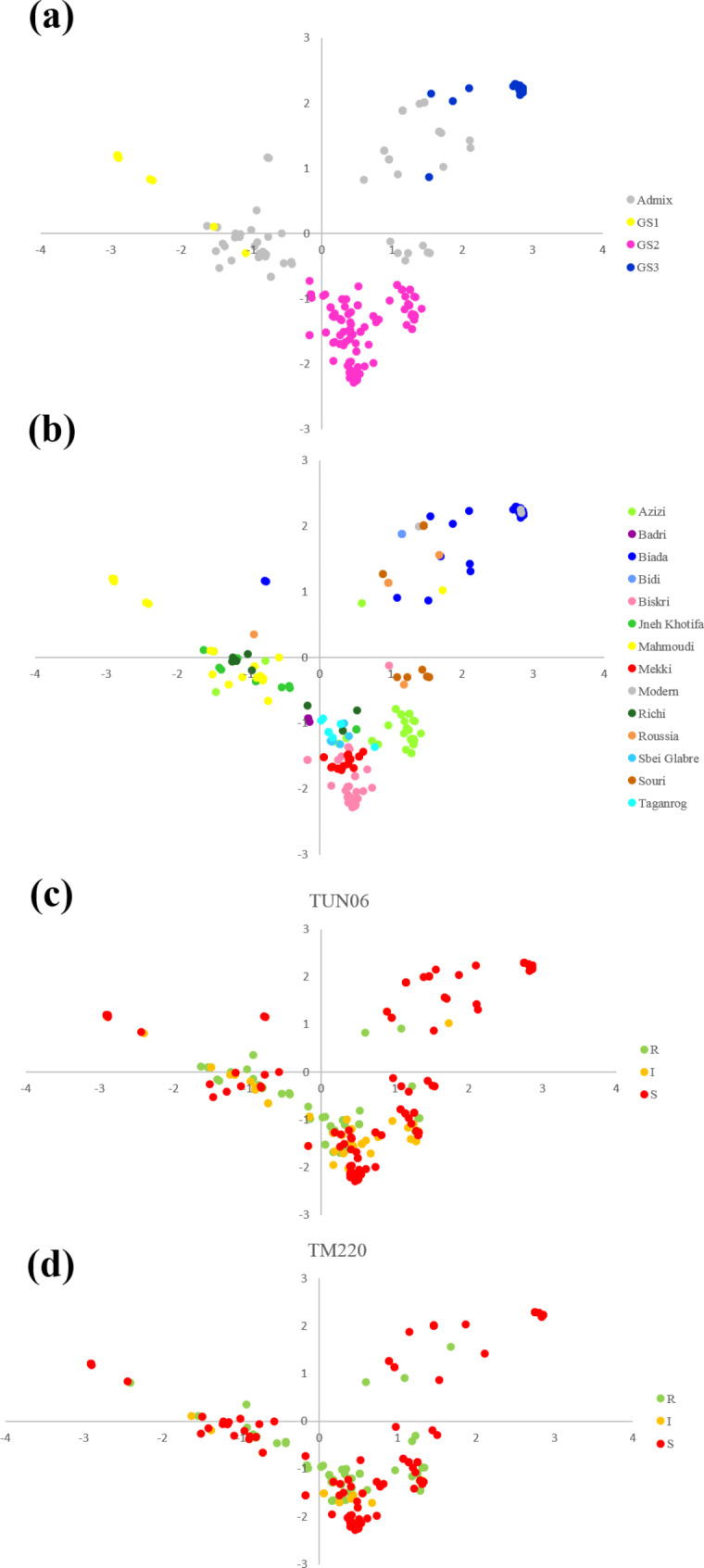
Principal component analysis plot of 366 Tunisian durum wheat accessions belonging to 13 landraces genotyped with 286 SNP markers under GenAlEx [101], color-coded by **(a)** genetic structure (GS1, GS2, GS3 and admixed genotypes) as identified by STRUCTURE [58] (for K = 3); **(b)** landraces denomination; and **(c)** resistance class to *Z. tritici* isolate Tun06 and **(d)** isolate TM220

PCAs were performed using the 286 SNPs on the 366 genotypes (Fig. 2). Axes 1 and 2 of the PCAs accounted for 24.33% and 16.54% of the total genetic variation, respectively. Figure 2a showed that PCA grouping corroborated the genetic structure inferred by STRUCTURE, pointing a clear differentiation between GS1, GS2 and GS3. Admixed genotypes were essentially distributed between GS1 and GS2 and between GS2 and GS3, reflecting ongoing hybridization and allele exchanges between these groups. Pairwise *F*st values also showed considerable genetic differentiations between GS1, GS2 and GS3 (Table 1a). The highest *F*st value (0.751) was observed between GS1 and GS3. *F*st values between GS1 and GS2 and between GS2 and GS3 were both around 0.400. Similarly, *Nm* indices between GS1 and GS2 and between GS2 and GS3 were close; while *Nm* between GS1 and GS3 was the lowest (0.078) reflecting an almost absence of genetic exchange between these two populations. The AMOVA (Table 2) was consistent with pairwise *F*st and *Nm* analysis revealing that the genetic variation between subpopulations (61%) was higher than the variation within subpopulation (39%).

**Table 1a:** Pairwise *Fs*t values (under the diagonal) and gene flow (*Nm*) (above the diagonal) calculated under GenAlEx 6.501 [101] between the genetic subpopulations as defined by STRUCTURE [65] (at K = 3)*

	GS1		GS2	GS3
**GS1**	-		0.408	0.078
**GS2**	0.380		-	0.424
**GS3**	0.762		0.371	-

*Admixed genotypes were not included in this analysis

**Table 1b:** Pairwise *Fs*t values (under the diagonal) and gene flow (*Nm*) (above the diagonal) calculated under GenAlEx 6.501 [101] between landraces*

	Azizi	Badri	Biada	Biskri	Jneh Khotifa	Mahmoudi	Mekki	Modern	Richi	Sbei Glabre	Taganrog
**Azizi**	-	0.298	0.349	0.592	0.362	0.215	0.468	0.318	0.646	0.4263	0.630
**Badri**	0.456	-	0.095	0.308	0.000	0.017	0.175	0.037	0.223	0.253	0.344
**Biada**	0.417	0.724	-	0.231	0.091	0.078	0.180	12.456	0.239	0.209	0.263
**Biskri**	0.297	0.448	0.520	-	0.235	0.217	0.439	0.210	0.625	0.456	0.609
**Jneh Khotifa**	0.409	1.000	0.734	0.516	-	0.025	0.209	0.035	0.213	0.177	0.286
**Mahmoudi**	0.538	0.935	0.761	0.536	0.911	-	0.152	0.054	0.205	0.197	0.261
**Mekki**	0.348	0.588	0.581	0.363	0.545	0.622	-	0.136	0.448	0.317	0.459
**Modern**	0.440	0.871	0.020	0.544	0.878	0.821	0.647	-	0.198	0.179	0.224
**Richi**	0.279	0.528	0.511	0.286	0.540	0.550	0.358	0.557	-	0.423	0.638
**Sbei Glabre**	0.370	0.497	0.545	0.354	0.586	0.559	0.441	0.582	0.372	-	1.109
**Taganrog**	0.284	0.421	0.488	0.291	0.467	0.489	0.353	0.527	0.281	0.184	-

*Admixed genotypes were not included in this analysis

**Table 2:** Analysis of molecular variance (AMOVA) of the Tunisian durum wheat accessions performed under GenAlEx 6.501 [101], by genetic subpopulation as defined by STRUCTURE [65] (at K = 3) and by landrace *

	Source	df	SS	MS	Est. Var.	%
**Genetic Subpopulations**	**Among**	2	33000.954	16500.477	199.754	61%
	**Within**	283	35911.928	126.898	126.897	39%
	**Total**	285	68912.881		326.652	100%
**Landraces**	**Among**	10	54241.466	5424.147	221.556	81%
	**Within**	275	14671.415	53.351	53.351	19%
	**Total**	285	68912.881		274.907	100%

**df**: degree of freedom; **SS**: Sum of Squares; **MS**: Mean Squares; **%**: pourcentage of variance; *Admixed genotypes were not included in this analysis

GS1 was solely composed by the landrace Mahmoudi. GS2 was composed of the landraces Azizi, Badri, Biskri, Jneh Khotifa, Mekki, Richi, Sbei glabre and Taganrog. GS3 was composed by the landrace Biada and two modern cultivars (Karim and Razzek). Twenty accessions of Mahmoudi, 11 of Jneh Khotifa, 8 of Richi, one Azizi and one Roussia accession were admixed between GS1 and GS2. Admixed genotypes between GS2 and GS3 included one accession of Azizi, 4 of Biada, 2 of Biskri, 5 of Souri, 5 of Roussia and 2 modern cultivars (Om Rabia andSalim). Finally, one accession of Mahmoudi, 2 of Biada, 8 of Bidi, 6 of Souri, 2 of Roussia and 2 modern cultivars (Nasr and Khiar) were admixed between GS1 and GS3 (Fig. 2b, Table S1).

In addition, population structure was investigated from K = 13 (corresponding to the number of landraces studied) to K = 20 (Table S2). At K = 13, landraces Azizi, Badri, Biada, Bidi, Jneh Khotifa, Mekki, Richi and Sbei glabre were assigned to separate genetic subpopulations. However, landraces Souri and Roussia were grouped in the same genetic subpopulation. Landraces Mahmoudi and Biskri were both divided into two genetic subpopulations. Taganrog landrace was entirely composed of admixed genotypes (between Sbei glabre, Jneh Khotifa, Azizi and Badri). The STRUCTURE assignment that matched exactly the landraces denomination was obtained for K = 15. Indeed, at K = 15, all 13 landraces were discriminated and assigned to genetically distinct subpopulations, except for Mahmoudi and Biskri, which were both divided into two genetic subpopulations as for K = 13. In fact, Mahmoudi subpopulations corresponded to two morphological types of Mahmoudi namely Mahmoudi-122 and Mahmoudi-986. Mahmoudi-122 had larger grain size and a relaxed spike comparing to Mahmoudi-986. Biskri subpopulations corresponded to two morphological types of Biskri namely Biskri-Ac1 and Biskri-glabre (Figure S1). Pairwise *F*st values based on landrace varied from 0.020 to 1 and most all the durum wheat landraces were genetically differentiated (Table 1b). Nonetheless, the lowest *F*st value (0.020) was observed between Biada and the modern varieties, followed by the *F*st value between Taganrog and Sbei glabre (0.184). These varieties shared almost the same agro-morphological characteristics. The lowest gene flow values were recorded between the modern varieties and all landraces, except for Biada. In addition, AMOVA (Table 2) showed that the genetic variation among landraces (81%) was higher than the variation within landraces (19%).

**Fig. 3:**
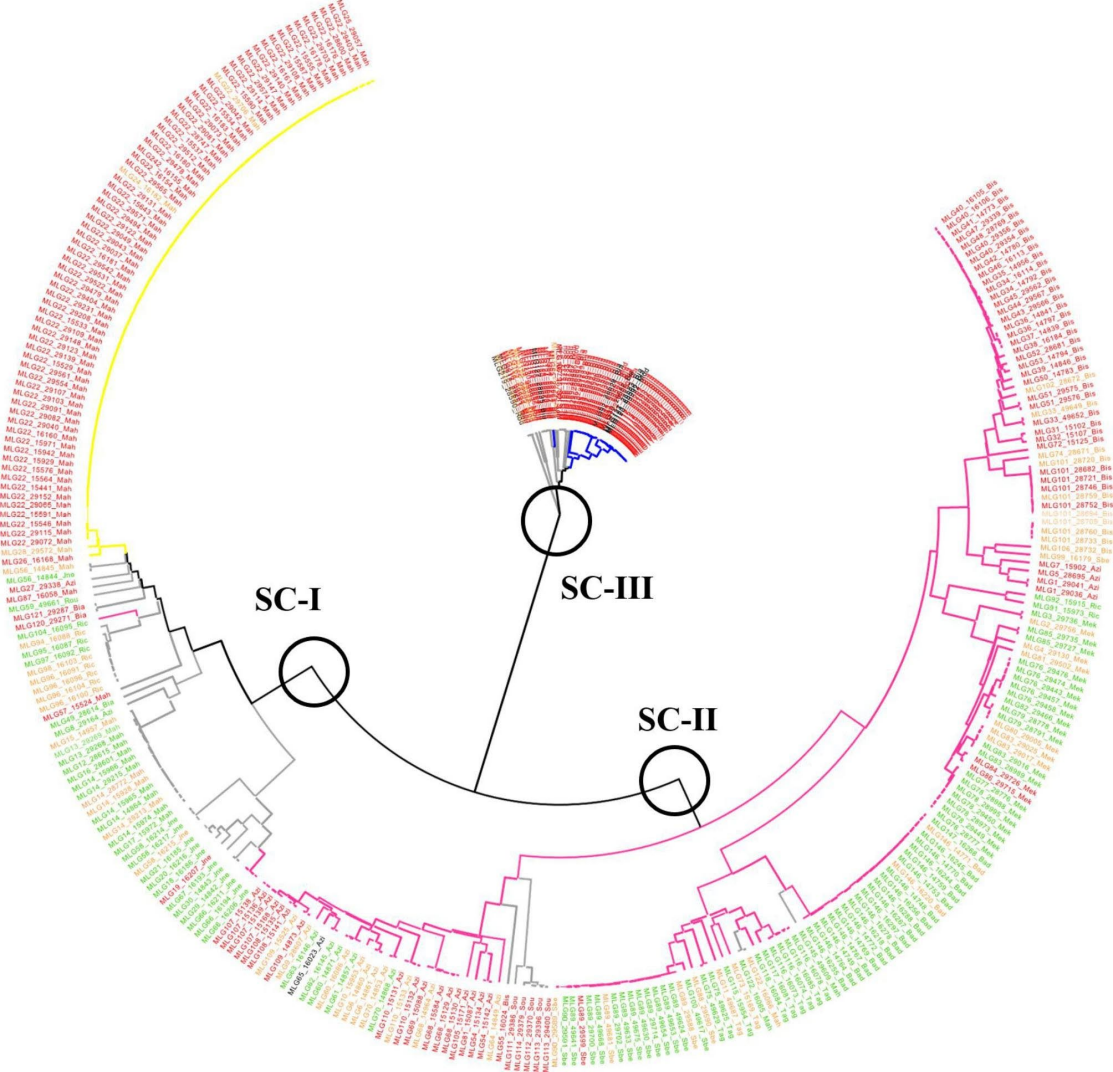
Unweighted Pair Group Method with Arithmetic Average (UPGMA) tree of 366 Tunisian durum wheat accessions genotyped with 286 SNP markers. Genotype names are labelled as listed in Table S3 and are color-coded by resistance class to *Z. tritici* isolate Tun06 . Branches are color-coded according to STRUCTURE results at K = 3 [65]. Three subclusters were identified and labelled SC-I, SC-II and SC-III.

The Unweighted Pair Group Method with Arithmetic Average (UPGMA) tree, generated with 286 SNPs data of 366 genotypes, differenciated three subclusters that were mainly in agreement with the genetic grouping defined by STRUCTURE (Fig. 3). Subcluster « SC-I » grouped all Mahmoudi GS1 and admixed genotypes, jointly with all GS2 and admixed genotypes of Jneh Khotifa, 2 Azizi admix, 3 Biada admix, one Roussia admix, 8 Richi admix and one Richi GS2. Subcluster « SC-II » included all Azizi, Badri, Biskri, Mekki, Sbei glabre, Taganrog and Richi GS2 genotypes, along with 5 Souri admix and 3 Mahmoudi admix. Subcluster « SC-III » grouped all GS3 genotypes notably Biada, together with 8 admixed genotypes of Bidi, 6 of Roussia, 6 of Souri and 2 of Mahmoudi.

Diversity analysis (Table 3) of the genetic subpopulations showed that GS2 presented higher diversity indices (*I* = 0.519; *He* = 0.353) than GS1 and GS3. The overall gene flow between the genetic subpopulations (*Nm* = 0.414) reflected a low gene exchange between the three groups. Diversity indices of the accessions based on their landrace nomenclature were the lowest for Badri and Bidi (*I* = 0.00; *He* = 0.00) and the highest for Roussia (*I* = 0.392; *He* = 0.259). These observations are consistent with pairwise *F*st values (Table 1b and AMOVA (Table 2) indicating that landraces were genetically distinct (low intra-population variability).

**Table 3:** Diversity indexes of 366 Tunisian durum wheat accessions grouped by genetic subpopulations as defined by STRUCTURE [65] (at K = 3) and by landraces

		N	Ne	*I*	*He*	%P	*Nm*
**Genetic Subpopulations**	**ADMIX**	80	1.729 (0.016)	0.588 (0.008)	0.405 (0.007)	99.65%	
	**GS1**	74	1.004 (0.001)	0.011 (0.002)	0.004 (0.001)	14.34%	
	**GS2**	165	1.623 (0.019)	0.519 (0.012)	0.353 (0.009)	95.10%	
	**GS3**	47	1.128 (0.018)	0.114 (0.013)	0.073 (0.009)	37.41%	
	**Total**	366	1.371 (0.012)	0.308 (0.009)	0.209 (0.006)	61.63% (21.19%)	0.414 (0.023)
**Landraces**	**Azizi**	39	1.370 (0.019)	0.368 (0.013)	0.233 (0.010)	89.86%	
	**Badri**	21	0.997 (0.003)	0.000 (0.000)	0.000 (0.000)	0.00%	
	**Biada**	51	1.194 (0.017)	0.218 (0.012)	0.127 (0.009)	77.97%	
	**Bidi**	8	1.000 (0.000)	0.000 (0.000)	0.000 (0.000)	0.00%	
	**Biskri**	44	1.243 (0.018)	0.255 (0.014)	0.156 (0.010)	75.87%	
	**Jneh Khotifa**	14	1.374 (0.023)	0.315 (0.018)	0.214 (0.012)	55.59%	
	**Mahmoudi**	96	1.175 (0.010)	0.233 (0.010)	0.131 (0.007)	94.06%	
	**Mekki**	28	1.164 (0.017)	0.168 (0.014)	0.104 (0.009)	41.96%	
	**Modern**	6	1.413 (0.022)	0.362 (0.017)	0.243 (0.012)	65.03%	
	**Richi**	11	1.237 (0.015)	0.255 (0.014)	0.160 (0.009)	57.69%	
	**Roussia**	7	1.430 (0.020)	0.392 (0.015)	0.259 (0.011)	73.78%	
	**Sbei Glabre**	20	1.096 (0.006)	0.150 (0.009)	0.080 (0.005)	56.64%	
	**Souri**	11	1.454 (0.027)	0.351 (0.018)	0.243 (0.013)	60.14%	
	**Taganrog**	10	1.320 (0.021)	0.295 (0.016)	0.194 (0.011)	58.39%	
	**Total**	366	1.248 (0.005)	0.240 (0.004)	0.153 (0.003)	57.64% (7.53%)	0.154 (0.005)

**N**: Number of accessions; **Ne**: Number of Effective Alleles; ***I***: Shannon’s Information Index; ***He***: Expected Heterozygosity; **P**: Percentage of Polymorphic Loci; ***Nm***: gene flow

### Distribution of the resistance to ***Zymoseptoria tritici*** among subpopulations

The 366 durum wheat accessions were evaluated for their resistance to two Tunisian *Z. tritici* isolates Tun06 and TM220. Based on disease scoring, the landraces were categorized into three classes of response to *Z. tritici* (R, I and S) as defined by Ouaja et al. [21]. Overall, 20%, 15.8% and 63.8% of the collection were R, I and S genotypes to isolate Tun06, respectively. In addition, 32.8%, 8.9% and 57.9% of the collection were R, I and S genotypes to isolate TM220, respectively.

The ANOVA (Table 4) revealed that the genetic subpopulation effect is highly significant, indicating that the variation in the responses to *Z. tritici* infection was consistent with the population genetic structure. However, a non-significant effect of isolates on the RAUDPC scores was revealed. Genetic subpopulations x isolates interaction was also non-significant. Accordingly, isolates Tun06 and TM220 did not significantly varied in their severity (RAUDPC scores) towards wheat accessions.

**Table 4:** Analyses of variance (ANOVA) performed under R 3.3.2 [97] on the Relative Area Under the Disease Progress Curve (RAUDPC) on the Tunisian durum wheat accessions genotyped with 286 SNPs. Main effects of genetic subpopulations, isolates and genetic subpopulations x isolates interaction were investigated

	df	SS	MS	F value	Pr (> F)	
**Genetic subpopulations**	2	128,937	64,468	110.509	< 2e-16	***
**Isolates**	1	751	751	1.287	0.257	
**Genetic subpopulations x Isolates**	2	765	383	0.656	0.520	
Residuals*	497	289,938	583			

**df**: degree of freedom; **SS**: Sum of Squares; **MS**: Mean Squares; *Admixed genotypes were not included in this analysis

The distribution of R, I and S classes by genetic subpopulation was uneven. However, the distribution of each class of resistance differed slightly between isolates Tun06 and TM220 (Fig. 2.c, d, Table S1). GS1 and GS3 were mainly formed by accessions of class S. Infact 93% and 85% of the accessions were susceptible to Tun06 within GS1 and GS3, respectively. For the isolate TM220, 64% and 34% of the accessions were susceptible within GS1 and GS3 respectively. Interestingly, 44% and 31% of resistant accessions to Tun06 belonged to GS2 and admixed genotypes with GS2, respectively. Similarly, 48% and 26% of the resistant to TM220 were GS2 and admixed genotypes with GS2 respectively. Genotypes with intermediate responses to Tun06 and TM220 isolates were also mainly among GS2 or admixed with GS2. While GS2 comprised less than 35% of susceptible accessions. About 44% of admixed genotypes (mostly GS1-GS2 and GS2-GS3) were susceptible.

The PCA of 366 Tunisian durum wheat accessions sorted by classes of response to isolate Tun06 (Fig. 2c) showed that resistant genotypes were mainly laying among GS2 and admixed genotypes GS1-GS2 and GS2-GS3. Susceptible genotypes were spread over the three subpopulations. UPGMA tree (Fig. 3) showed that the three subclusters grouped genotypes of different resistance classes. All susceptible Mahmoudi GS1 genotypes and mostly resistant admixed genotypes between GS1 and GS2 (Mahmoudi, Jneh Khotifa, Badri and Richi) were in SC-I. SC-II was essentially composed of resistant GS2 landraces (Taganrog, Sbei glabre, Richi, Mekki, Badri and Azizi). However, 100% of Biskri accessions, 54% of Azizi, 7% of Mekki, 5% of Sbei glabre and 45% of Souri admix were within SC-II and showed susceptibility. SC-III is entirely composed of susceptible genotypes of Biada GS3, Biada admix, Bidi admix, Souri admix and Roussia admix.

## Discussion

Old durum wheat germplasm represents a precious genetic heritage. Understanding the genetic and phenotypic structure of old local landraces will help retracing their life history, resistance and durability of use. Durum wheat population structure study also help deciphering new sources of resistance to cope with challenging abiotic and biotic stresses, notably STB, one of the most devastating fungal disease on durum wheat crop. In the present study, we genotyped 366 Tunisian durum wheat accessions belonging to 13 old Tunisian landraces [22], collected from three central and two southern regions in Tunisia, using 286 SNPs derived from a High-density 90 K wheat SNP array [67]. This study revealed the population structure and the genetic distribution of the resistance to *Z. tritici* in the Tunisian durum wheat landraces.

### Identification of a population structure in the tunisian durum wheat landraces related to their potential introduction pathways

The genetic structure of 366 durum wheat accessions was investigated using 286 SNPs. Three major genetic supbpopulations (K = 3: GS1, GS2 and GS3) were obtained under STRUCTURE [65]. At K = 15, we were able to attribute each landrace to a distinct genetic group, with the exception of Mahmoudi and Biskri landraces that were both divided into two groups. This result is in agreement with our previous study [22], where 8 out of 11 Tunisian durum wheat landraces corresponded to distinct genetic groups using 10 SSR markers. AMOVA analysis showed a high genetic variability (61%) between subpopulations, suggesting that the three subpopulations were derived from different gene pools. This is consistent with previous studies of tetraploid wheats [2, 68] and barley [69] showing that local landraces were derived from multiple ancestral populations and had reticulated phylogenetic relationships. However, several other durum wheat landrace studies reported a higher or an equal genetic variation whitin population than among population [30, 70, 71]. Soriano et al. [11] detected only 13% of genetic varibility between four genetic subpopulations that were tracing distinct geographical pattern of the Mediterranean durum wheat germplasm. In this study, GS1 and GS3 were composed essentially with Mahmoudi and Biada landrace, respectively. These landraces have preferred specific agro-morphological traits recognized by the farmers, selected and multiplied over time. The formation of distinct domesticated gene pools were also reported for several other crops such as common bean, which underwent parallel evolution and spread further through the development of landraces with distinct characteristics and specific adaptations [72–75].

Informations about putative origins and years of introduction in Tunisia of the 13 herein studied landraces, along with their agro-morphological characteristics as described by Ouaja et al. [22], were analyzed to explain their inferred genetic structure. In fact, the grouping pattern of accessions appear to be associated, to some extent, with the geographical pattern of the landraces. Indeed, Slim et al. [20] highlighted a strong North-South stratification in Tunisian durum wheat landraces with the prevalence of modern cultivars in the North versus landraces, grown by small-holder farmers under low-input traditional agrosystems in the marginal areas of the Center and South However, other durum wheat landrace studies showed a genetic clustering irrespective of their geographical origin, underlying the presence of plant material exchanges that could have reduced the genetic differentiation [41]. In this study, the subpopulation GS2 mainly included landraces of North African and East Mediterranean origins. GS2 includes landrace Mekki from Morocco, landrace Taganrog from Southern Russia, landraces Azizi, Jneh Khotifa and Sbei glabre that were considered as local populations and Richi which was reported as foreign [17, 33, 76]. Robbana et al. [13] also reported that Tunisian landraces were genetically associated to North African landraces. However, using a set of DArtSeq markers to describe the genetic diversity of Tunisian landraces, Robbana et al. [13] reported that Jneh Zarzoura, a close relative to Jneh Khotifa [17] clustered distinctly with accessions from Jordan. Moreover, local landraces names have been traditionally selected by natives, generally according to morphological features or locality, and are often consciously used by farmers for management, selection or exchanges [14, 77]. Sahri et al. [77] particularly highlighted the significance of variety name, which have largely influenced the structure and evolutionary dynamics of durum wheat in Morocco. Therefore, nominal analogies were frequently reported between landraces of different Mediterranean regions, probably reflecting trade and migration of the same landraces around the Mediterranean basin. Xynias et al. [6] reported that the Italian cultivar « Senatore Capelli » was selected in 1915 from the local North African landrace « Jean Retifah » which was very prominent and marked the cultivation of durum wheat in Italy. The landrace « Jean Retifah » must be the known Jneh Khotifa in Tunisia [14, 17]. In addition, Soriano et al. [11] studied the structure of durum wheat landraces from 21 Mediterranean countries, using SSR markers, and reported two Italian landraces named « Hymera » and « Aziziah » that were associated to eastern Mediterranean genetic group. Knowing that Tunisia was the former bread basket of the Roman Empire [78, 79], these latters could be the landraces known Hmira and Azizi in Tunisia [14, 17, 20, 22], suggesting though that Azizi landrace may also have an eastern origin. A recent study on 170 durum wheat landraces from 24 Mediterranean countries revealed that more than 23% of Tunisian landraces were from eastern mediterraneen countries [40]. Boeuf [14] mentioned that the landraces of North Africa had dominant characters, specific to Abyssinian wheats, such as red, purple or black spikes, pubescent glumes and red or dark-colored grains. These characters were totally absent and unknown in Europe. Herein it was noted that landraces of GS2, with the exception of Biskri, shared features of spikes and grains [22] corresponding to the Abyssinian wheats as Bœuf [14] reported.

Furthermore, according to Bœuf [14], the geographical expansion and domestication of wheats from Abyssinia enhanced the accumulation of recessive characters, in particular white spikes, hairless glumes and light-colored grains widely adopted in Europe. During crop domestication process, several changes were induced for major morphological, structural and functional traits associated with adaptation and cultivation in order to meet human needs, as reported for the common bean ) [72]. Bœuf [14] mentioned that the whiteness of the spike and light-colored grains were among the most sought after and preferred criteria in wheat by European farmers during commercial trade in North Africa. So far, these traits are characteristics of the landraces Mahmoudi (GS1), Biskri (GS2), Biada and Bidi (GS3) [22]. Landraces Biskri, Biada and Bidi were introduced in Tunisia from Algeria, while Mahmoudi was considered as a local landrace population with various reported origins including Algeria and Italy. Another example reflecting nominal analogy between Mediterranean landraces is the Tunisian landrace Biada and the Spanish landraces « Blancal » and « Blanco de Baleares » meaning, among others, the white wheats [17, 22, 80], thus indicating that such phenotypic characteristic was probably derived from western Mediterranean. Based on these findings/statements, although the majority of Tunisian landraces are North Africa, we suggest that subpopulations GS1 and GS3 were introduced to North Africa and particularly to Tunisia from Europe/western Mediterranean, unlike subpopulation GS2 which may be originated from the Middle East. Ben Krima et al. [30] also agree that the combination of both genetic and agro-morphological approches are essential for retracing the history, origin and dynamic lifestory of Tunisian durum wheat landraces. In the same context, Moragues et al. [9] highlighted two dispersal pathways of the Mediterranean durum wheat landraces which had contributed to the divergence of these landraces into distinct genetic groups following their adaptation to different local environments. A first pathway through the North-East of the Mediterranean basin to Europe, and a second pathway through the South of the Mediterranean basin to North Africa reaching the Iberian Peninsula.

### Genetic distribution of the resistance of ***Z. tritici*** in the tunisian durum wheat populations

Tunisian durum wheat landraces have been reported to exhibit valuable sources of resistance to STB, useful to include in breeding programs and to develop varieties with durable and broad spectrum of resistance [21, 63, 64]. In the present study, the 366 genotyped durum wheat accessions were also screened for *Z. tritici* resistance, under field conditions, using two *Z. tritici* isolates Tun06 and TM220 collected from two Tunisian regions, Bizerte and Manouba, respectively. The analysis of variance showed a non-significant variation between isolates Tun06 and TM220 towards the durum wheat accessions.This result agrees with Ferjaoui et al. [63] findings, detecting only two virulence profils among 55 Tunisian *Z. tritici* isolates screened at seedling stage.

Overall, 60% of the accessions were susceptible, showing that Tun06 and TM220 isolates were virulent on the majority of the accessions, which reflect the adaptation of *Z. tritici* virulence to durum wheat landraces in Tunisia. Likewise, Ouaja et al. [21] suggested that the Tunisian *Z. tritici* isolate Tun06 still preserve virulences against old durum wheat landraces even though they are currently marginally grown in wheat production areas in Tunisia. In addition, several studies reported that *Z. tritici* undergoes frequent sexual reproduction on durum wheat in Tunisia [81, 82]. In fact, sexual reproduction plays a key role in the evolution of pathogenicity traits, including virulence and aggressiveness [83], enabling the fungus to quickly evolve and circumvent the resistance genes by creating new combinations of alleles and, in combination with the asexual reproduction allowing frequent generation of the new genotypes [49, 82]. Nevertheless, about 27% of the collection was resistant, suggesting that Tunisian durum wheat landraces still carry effective STB resistance genes.

The analysis of variance showed a large and significant variation between GS1, GS2 and GS3 regarding the resistance to isolate Tun06. Subpopulations GS1 (composed of Mahmoudi accessions) and GS3 (Biada accessions) showed higher frequencies of susceptible responses than GS2. The distribution of the resistance within each landrace observed in the UPGMA tree, indicate that the resistance relies on the landrace instead of the genetic structure (K = 3), as GS2 grouped both resistant and susceptible genotypes. These results also indicate that although landraces of GS2 formed an invidivualized genetic group, they may harbor combination of resistance genes that differ in nature, number, structure, chromosomal localisations and type of interaction that still need to be depicted and revealed by a genome wide association study (GWAS).

The susceptibility observed in the GS1, GS3, Biskri, and Azizi of GS2 genotypes can be attributed to their widespread use and commercial share, leading to extensive cultivation, particularly in northern Tunisia. Over time, these landraces have lost their resistance to the disease [14, 17]. In fact, *Z. tritici*, the causative agent of the disease, is predominantly prevalent in the northern and northwestern regions of Tunisia where sub-humid zones become significant hotspots for STB, exhibiting high levels of infection [59, 60, 84]. As a result of the rapid adaptation of *Z. tritici* isolates, the landraces extensively grown in the northern zones became susceptible to the disease [49, 56, 61, 85]. Moreover, rapid adaptation of *Z. tritici* to landraces from western mediterannen origin (GS1 and GS3) could be facilitated by domestication process mainly accompanied by a strong reduction in genetic diversity and/or high levels of gene loss compared to wild ancestors or wild gene pool [86–88]. These events reduced the adaptation of cultivated wheat to erratic environmental variations, where wild traits show much greater fitness over domesticated ones [75]. This senario might be consistent with the significant variability of the resistance to *Z. tritici* observed between subpopulations in the present study, suggesting that resistance (R) genes of the three subpopulations may have evolved divergently and crucially under a combination of environmental and human pressures. Therefore, we hypothesize that major genes conferring specific resistance (as dominant characters) have undergone modifications and alterations by mutations during the geographic expansion and with the wide exchange network of durum wheat landraces among Mediterranean regions, which resulted in the loss of dominant R genes/alleles and the spread of susceptibility as observed within landraces of subpopulations GS1 and GS3. Alternatively, the results suggest that landraces of GS1 and GS3 were initially susceptible to *Z. tritici* when introduced to Tunisia. Nevertheless, unlike GS1 and GS3, most landraces of GS2 (except for Biskri and Azizi landraces) were resistant because they had recourse to different geographical patterns/pathways as they were local or directly derived from an eastern origin and did not pass via Europe. Western Europe farmers were among the first to create and adopt modern methods of plant breeding and exert wheat genetic improvement, involving direct selection for homogeneous material, thus, indirectly reducing the variability of the genetic sources of resistance or even unintentionally selecting for recessive genes [14, 89, 90]. In contrast, smallholder farmers in North Africa have been preserving the local durum wheat diversity with on-farm conservation practices over generations [14, 17, 22, 77].

In this study, 22% of the accessions were admixed, among which 51% of admixed genotypes between GS2 and GS1 and 24% admixed between GS2 and GS3. Admixtures occurs mainly by gene flows, through the frequent introduction of new genotypes into fields and seed exchange network within and between farmer communities [2, 11, 16]. In fact, *Nm* between GS1 and GS2 and between GS2 and GS3 were both around 0.4. All the resistant accessions of Mahmoudi were admixed between GS1 and GS2 and all the resistant accessions of Biada, Souri and Roussia were admixed between GS3 and GS2; suggesting that resistant landraces in GS2 were probably the sources transmitting resistance to *Z. tritici* via admixture. Indeed, resistant Mahmoudi genotypes may have acquired their resistance from Jneh Khotifa, as they appeared phylogenetically close in the UPGMA tree. The UPGMA tree also showed that modern varieties are genetically close to Biada (GS3), indicating that modern varieties have been selected for certain agro-morphological and phenological characteristics of GS3, such as white and short spikes, short plant and precocity. In addition, modern varieties with a genome derived from GS3 or GS1 or admixed, have been selected from a susceptible background to *Z. tritici*. It is therefore necessary to re-direct the breeding programs towards another genepool presenting resistance to *Z. tritici*, for example by developping marker-assisted selection to introgress the resistance to *Z. tritici* from GS2. On the other hand, admixture may have also caused loss of resistance as some admixed Jneh Khotifa and Richi accessions were susceptible to Tun06. This result indicated that admixture between genetically distinct landraces/populations and frequently recurring gene exchanges (or gametic association between gene loci) [91], may have elicited susceptibility within initially resistant accessions throughout an alteration or loss of the resistance genes/alleles.

## Conclusion

The present study revealed that Tunisian durum wheat life history of resistance to STB involve the interaction of miscellaneous factors, including the landrace genetic structure and introductory pathways, the local commercial share defining the geographic and regional distribution of the landraces and the occurrence of admixtures within these landraces. In fact, landraces were subjected to genetic differentiation in time and space during their introductory pathways in the Mediterranean area, their adaptation to specific environments and the Human selection pressure/domestication, contributing to their divergence in their resistance spectra. In addition, the regional distribution of the landraces across Tunisia influenced the evolutionary history of resistance genes under different climatic constraints in relation to hotspots of STB disease. Admixtures also represent one of the main driver factors of the resistance to *Z. tritici* involving old or ongoing exchanges that contributed to the introgression and/or the loss of resistance genes. Our results suggest the presence of diverse resistance sources towards two *Z. tritici* isolates Tun06 and TM220 in the Tunisian durum wheat landraces, which implies the need for more in-depth research to investigate and characterize the related resistance genes. Continuous identification of new sources of resistance to STB is required for the development of wheat cultivars with sustainable field resistance. Therefore, old local durum wheat landraces can play an important role as donor of resistance genes in breeding programs. In addition, further investigation and study of virulence patterns in *Z. tritici* populations in Tunisia will provide new insights and understanding of the *Z. tritici*-durum wheat interactions and trace their co-evolution.

## Methods

### Wheat accessions

A set of 375 durum wheat accessions was used in this study. Accessions were collected by the National Gene Bank (BNG) from four regions in Central (the Sahel and Kairoun) and southern (Gabes and Medenine) Tunisia (Table S3). Accessions were morphologically characterized, identified and classified into 13 landrace-populations namely Mahmoudi, Biada, Bidi, Biskri, Azizi, Badri, Mekki, Jneh Khotifa, Sbei glabre, Taganrog, Richi, Souri and Roussia as described by Ouaja et al. [22]. Information about the origin and year of introduction and selection of these landraces are depicted in Table S4.

### Field trials and ***Z. tritici*** isolates

Durum wheat landraces were screened for their resistance to *Z. tritici* at the adult plant stage in the experimental station of CRRGC Beja in northwest Tunisia. Inoculation assays were performed using two durum wheat-derived *Z. tritici* isolates on separate field experimental plots; notably the well-characterized and virulent reference isolate Tun06 (also reported as TunBz-1) sampled from the Bizerte region of Tunisia in 2006 and the isolate TM220 collected from the Chili landrace cultivated in Lansarine region of Tunisia in 2014 [21, 61, 92, 93]. The virulence patterns of TM220 and Tun06 isolates were previously assessed at seedling stage on 21 old durum wheat accessions set and revealed that TM220 was virulent on 8 accessions comparing to isolate Tun06 which was avirulent (Thierry Marcel personnel com.). Tun06- and TM220 field trials were realized as reported by Ouaja et al. [21] and followed an Augmented Randomized Complete Block Design (ARCBD), including 6 blocks spaced apart of 1 m. Blocks were 1 m width linearly drilled and accessions were sown 20 cm spaced apart. Each block was composed of 70 accessions and 7 checks. The checks included six susceptible to moderately resistant modern durum wheat cultivars “Karim, Khiar, Om Rabia, Salim, Maali and Nasr” [60, 61]. Durum wheat landraces were evaluated for resistance to Tun06 isolate during two consecutive growing seasons (2015–2016 and 2016–2017), and for resistance to isolate TM220 on the growing season of 2016–2017.

### Inoculation and screening for resistance to ***Z. tritici***

Tun06 and TM220 isolates were maintained frozen at -80 °C. A preculture of the inoculum was prepared by shaking *Z. tritici* isolates (at 100 rpm/ 25 °C) for six to seven days, in 100 ml yeast glucose liquid medium (30 g glucose, 10 g yeast per liter demineralized water). The produced spore suspensions of Tun06 and TM220 were subsequently transferred to 500 ml yeast glucose liquid media and were incubated under the aforementioned conditions to provide sufficient inoculum for the field trials. Spores of both isolates were collected after overnight settling in static cultures, concentrated by decanting the supernatant medium, and were adjusted to 10^6^ spore/ml. Accessions in all experimental plots were inoculated twice, at the three-leaf stage (approximately Growing stage 21) and at the stem elongation stage (approximately Growing stage 37) [94] as described by Ouaja et al. [21].

The disease severity was evaluated by estimating pycnidia coveragepercentages which correspond to sporulating area. The same leaf layer (F3-F4) of each accession in all experimental plots was assessed for STB resistance, at three time points for isolate Tun06 and at two time points for isolate TM220. Tun06-disease scores were assessed at 18, 38 and 46 days post the second inoculation (dpi) during 2015–2016 growing season, and at 15, 35 and 53 dpi during 2016–2017 growing season. TM220-disease scores were assessed at 20 and 40 dpi during 2016–2017 growing season. Screening data were used to calculate the Area Under the Disease Progress Curve (AUDPC) and the Relative Area Under the Disease Progress Curve (RAUDPC) for quantitative analyses of the temporal differences in disease progress as detailed by Ouaja et al. [21].

Three classes of response to *Z. tritici* at the adult plant stage, defined by Ouaja et al. [21], were considered in this study. The resistant class (R) include accessions having pycnidia score < 25%, while the susceptible class (S) include accessions showing > 47% of pycnidia on infected leaves. Accessions with pycnidia score ranging between 25% and 46% constitute the class of intermediate (I). Two and 63 accessions were not scored for STB resistancefor Tun06 and TM220, respectively. The variation of classes of response R, I and S between the isolates Tun06 and TM220 is shown in Table S7.

### Genotyping and data analysis

A total of 20,279 polymorphic SNPs were generated by Illumina sequencing 375 Tunisian durum wheat accessions using a High-density 90 K wheat SNP array (iSelect, San Diego, USA) [67], among which 13,998 (~ 70%) were mapped in the consensus durum wheat genetic map [95]. The filtered SNPs had < 5% of missing data and a minor allele frequency of 5% were filtered. From these markers, 286 SNPs covering the entire genome with 12 to 23 SNPs per chromosome of 10 cM apart and with a PIC > 0.3, were selected to perform the analysis in the present study (Table S5). PIC values were calculated by determining the frequency of alleles per locus as Powell et al. [96] (Table S6). Genotypes with > 10% of missing data out of the 286 SNPs were eliminated, and 366 genotypes were included in the rest of the analysis. Based on 286 SNP data generated for 366 accessions, 147 multilocus genotypes (MLG) were identified with R 3.3.2 [97] (Table S1). According to genotype accumulation curves (**Figure S2**), performed under R 3.3.2 [97], the 286 SNPs (**Figure S2a**) provide enough discrimination between the indiviuduals than the 13,998 SNPs (**Figure S2b**) and have a good power of resolution to study the population structure and genetic diversity of the Tunisian durum wheat.

The genotypic data based on the 286 selected SNP markers were used to study the population structure of the Tunisian durum wheat accessions with the model-based clustering algorithm STRUCTURE 2.3.4 [65]. STRUCTURE program was run on 147 MLG with K values between 1 and 20, by applying 10 independent runs for each value of K, 100,000 burnins and 100,000 Markov Chain Monte Carlo (MCMC) repetitions. The optimal number of genetic subpopulations was determined using the mean posterior probability (ln P(D)) value per cluster (K) and the delta-K method of ln P(D) under STRUCTURE harvester 0.6.9.4 [66]. Population structure was investigated at the optimal K as well as at K ranging from 13 to 20 in order to assertain the genetic grouping according to landrace denomination. Individuals presenting a membership coeficient less than 0.7 to any genetic subpopulation were considered as admixed genotypes. To study the phylogenetic relationships between accessions/landraces, an Unweighted Pair Group Method with Arithmetic Average (UPGMA) tree was created using Nei standard genetic distance [98]. A bootstrap analysis was performed with the program Populations 1.2.32 [99] and branch support values were estimated using 1000 bootstrap randomizations. A consensus tree was generated and viewed using TreeView 1.6.6 [100].

In addition, pairwise *F*st and gene flow (*Nm*) coefficients were calculated with GenAlEx 6.501 [101] by genetic subpopulations and by landraces (based on their nomenclature). An analysis of molecular variance (AMOVA) were also carried out using GenAlEx 6.501 [101] in order to investigate the significance of genetic subpopulations differentiation as well as genetic differentiation between and among landraces. A Principal Components Analysis (PCAs) on the genotypic data was performed under GenAlEx 6.501 [101]. This multivariate analysis was completed to visualize the structure of the 366 durum wheat accessions sorted by genetic subpopulations (as defined by STRUCTURE), by landrace denomination [22] and by STB resistance class to Tun06 (R, I and S). Furthermore, genetic diversity indices (Ne, *I*, *He*, P) were calculated by genetic subpopulation and by landrace [101].

An analyses of variance (ANOVA) were carried out under R 3.3.2 [97] to determine the effect of genetic subpopulations and isolates on RAUDPC scores. Genetic subpopulations x isolates interaction were also investigated.

## Electronic supplementary material

Below is the link to the electronic supplementary material.

Supplementary Material 1

## Data Availability

The seeds of each accession can be requested from the National Gene Bank of Tunisia GRIN-Global repository (http://tn-grin.nat.tn) according to their NGBTUN ID under the following web link http://www.tn-grin.nat.tn/gringlobal/search. The data sets supporting the results of this article are included in this manuscript in its additional information files. Genotyping data are available in an excel sheet ‘Table S5’ with a corresponding SNP code.

## Abbreviations

BNG: National Gene Bank of Tunisia
STB: Septoria tritici blotch
SNP: Single Nucloetoide Polymorphic marker
UPGMA: Unweighted Pair Group Method with Arithmetic Average
SC: Genetic subcluster; GS = genetic subpopulation
